# First-time description of MHC diversity in Straight Egyptian Arabian horses using immune-linked microsatellite markers

**DOI:** 10.3389/fimmu.2026.1859977

**Published:** 2026-08-17

**Authors:** Abdullah Saleh A. Alkhamees, Jessika-M. V. Cavalleri, Sabine E. Hammer

**Affiliations:** 1 Immunology Unit, Centre of Pathobiology, Department of Biological Sciences and Pathobiology, University of Veterinary Medicine Vienna, Vienna, Austria; 2 Equine Internal Medicine, Clinical Centre for Equine Health and Research, Clinical Department of Small Animals and Horses, University of Veterinary Medicine Vienna, Vienna, Austria

**Keywords:** ELA, equine leukocyte antigen, major histocompatibility complex, MHC diversity, microsatellite analysis

## Abstract

Understanding the immunogenetic structure of the major histocompatibility complex (MHC) in Arabian horses helps to clarify the population health structure-related phenotypes of this historically influential breed. Straight Egyptian (SE) Arabian horses, which fall under *Equus ferus caballus*, are of global value but are limited in number, raising concerns about their distribution and the genetic diversity associated with immunity. In this study, we examined 57 Egyptian Arabian horses from two breeding programs: one in Germany (AR-DE) and the other in Qatar (AR-QA). Sixteen polymorphic intra-MHC microsatellite markers were used to cover classes I, II, and III. Population genetic calculations were then performed to determine allelic richness, heterozygosity, fixation indices, and departures from the Hardy–Weinberg equilibrium (HWE) after the fragment length analysis. Despite the small population size, the diversity and allelic richness were remarkable. The range of alleles per locus was 4–12, with a mean of 7.88 in all horses. Observed heterozygosity (Ho) was between 0.070 and 0.860, while the expected heterozygosity (He) was between 0.542 and 0.877, suggestive of heterozygote deficiency at several loci, based on a mean fixation index (*F*) of 0.226. Polymorphism information content (PIC) was from average to highly informative, with a mean of 0.672. When the population was divided into two groups, AR-DE and AR-QA, both showed overlapping genetic patterns. However, in the AR-DE group, a higher mean was observed in Ho (0.604) and a lower mean in *F* (0.061). In the AR-QA group, there were more genetic loci diverging from HWE, and the shortage was also more evident in the mean *F* values (0.142). Strong heterozygote deficits in loci such as TKY3324 and CZM004 in both populations likely reflect locus-specific selection or population structure. Overall, the results present statistical evidence of MHC-associated genetic diversity in SE horses that equals or exceeds levels observed in other Arabian horse breeds or in geographically isolated populations. The Ho pattern and HWE deviations indicate possible selection within the MHC and demographic history. This study could provide a firm foundation for the genetic immunology of Egyptian horses and support the wider use of microsatellites to assess histocompatibility diversity in equine populations.

## 1 Introduction

Arabian horses have been documented in the Middle East and Egypt since the second millennium BC (1, 2). Their presence and domestication are evidenced by fossils and stone inscriptions, indicating that they were domesticated and preserved by humans (1, 2). The Arabs have preserved and documented the lineages of these horses, classifying them into families such as Dahman, Saqlawi, Kuhaylan, and Abayyan, a practice that continues to this day (1, 3–5). Despite the historical importance of Straight Egyptian (SE) Arabian horses, there are not enough studies examining their genetic diversity and the characteristics of key genetic loci associated with immunity, especially the major histocompatibility complex (MHC). This study seeks to highlight the variation in MHC in SE Arabian horses using highly polymorphic intra-MHC microsatellites. This may contribute to a more comprehensive understanding of genetic diversity within this lineage.

MHC genes demonstrated the most polymorphic loci in the vertebrate genome (6). The development of autoimmune disorders and determination of host resistance or vulnerability to pathogens might be affected by the variations within the MHC (6–8). This concept is well illustrated by the association of HLA-B53 with resistance to severe malaria in West Africa (9, 10). Horses constitute a domestic animal species with a long history of intensive selective breeding and continuous exposure to diverse pathogens. From an immunological perspective, heterozygosity in MHC-associated markers is important. Individuals with high heterozygosity carry a wider range of MHC phenotypes, potentially increasing the range of peptides that can be presented to T cells. Conversely, a significant lack of heterozygosity leads to a decrease in the number of phenotypes. These phenotypes are particularly important for selectively managed, small-population horse breeds, where breeding decisions influence the maintenance of immunologically related genetic variation.

**Figure 1:**
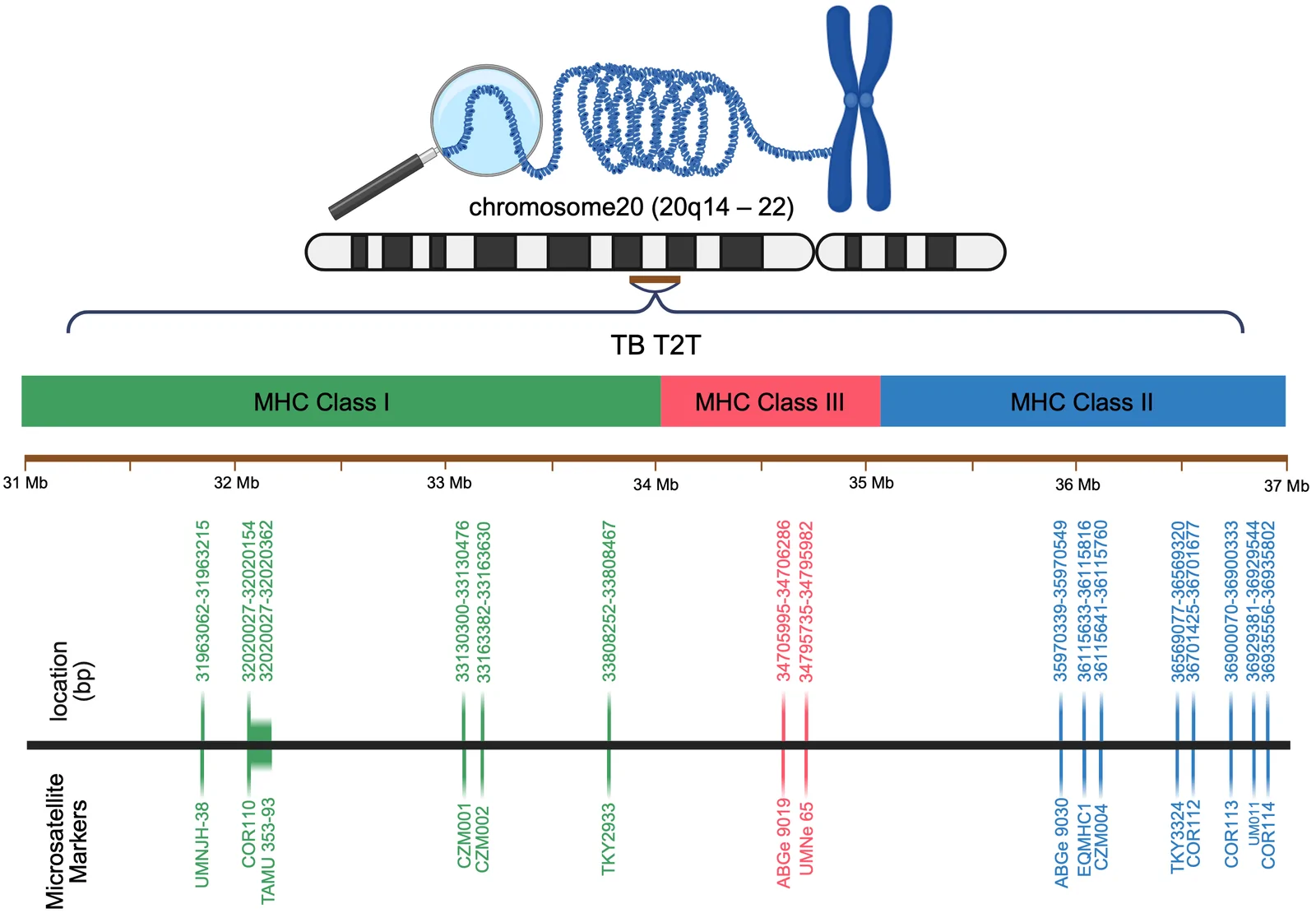
Map of intra-MHC microsatellites used in the study. Genomic location of horse intra-MHC microsatellites. Chromosome 20 from the TB-T2T (assembly no. GCA_041296265.1) is displayed. MHC location is arranged into three subgroups: class I, class II, and class III. The 16 microsatellite markers used in this study are listed below the line (UMNJH-38, etc.; see **Table 1**).

Selective breeding practices are recognized as a major driver of reduced genetic diversity in domesticated animals including horses (11). In contrast, tamed animals are subjected to intentional artificial selection (12, 13). Consequently, beyond pathogen- and fitness-related pressures, the MHC gene pool in domesticated animals may also be shaped by human-driven preferences for distinct traits (12–14). The MHC in horses is commonly referred to as the equine leukocyte antigen (ELA) system, which is located on chromosome 20 (20q14–22) (15, 16). The MHCI and MHCII regions comprise a complex array of highly polymorphic and polygenic loci, which, owing to the technical limitations of earlier molecular technologies, have been only partially characterized in non-model yet economically important species such as the horse (17). International workshops conducted in the 1980s validated 19 serological particularities that delineated the antigenic products of the ELA system (18). The association between MHC variation and immune-mediated diseases (e.g., insect bite hypersensitivity) (19–21), equine sarcoids (22), and equine recurrent uveitis (23) has been reported. The horse genome sequence TB-T2T refers to the thoroughbred telomere-to-telomere genome assembly. The genomic sequence for this RefSeq record is from the whole-genome assembly released by the University of Kentucky on 16 August 2024 (24). The original whole-genome shotgun project has the accession number JBFNWK000000000.1 (**Figure 1**).

It is well known that the economical and suitable usage of the serological lymphocyte microcytotoxicity test has been established. Nevertheless, it always detects a broad MHC haplotype group, making it neither suitable nor precise enough for the comparison studies of MHC haplotypes. Moreover, it has another limitation regarding the restricted range of antibodies; thus, it has achieved the least clarification for more than 35 years, whereas genotyping using microsatellite markers represents an efficient and low-cost technique for analyzing the diversity of complex genomic regions, like the MHC, within a large range of animal species (11). The linkage map of the equine genome using molecular genetic analysis since the 1990s resulted from polymorphic microsatellite markers (25). The maintenance of selective breeding influences the phenotypic as well as the characteristics of modern horses and their genomic map diversity of MHC genes (26). There are similarities among serotypes and microsatellite haplotypes in specific breeds, indicating that the profiling of intra-MHC microsatellite can accurately be used as an MHC genotyping assessment in both population and family surveys, notably for thoroughbreds and Standardbreds (27). The finding of more intra-MHC microsatellite loci in the horse (28) implements the extension of the well-known microsatellite markers (29).

The polymorphism of MHC genes has been evidenced in many published reports, including different horse breeds of the Equidae family (21, 29–43). However, the allelic repertoire of the equine MHC is still elusive or slightly authentic as reported by the IPD-MHC database (https://www.ebi.ac.uk/ipd/mhc/group/ELA/, accessed on 15 September 2022); sequence information is currently available for just 48 MHCI alleles, 5 ELA-DRA, 20 ELA-DRB (representing DRB1–3 loci), 13 ELA-DQA (DQA1–3 loci), and 15 ELA-DQB alleles (of DQB1–3 loci) (17).

The laborious and expensive techniques currently used for sequence-based typing of mammalian species as well as humans showed that the alternative, namely, genotyping of microsatellite markers of MHC, is an adequate assessment of genetic diversity for large-scale cohorts. Therefore, it has been broadly used to analyze population structure, countify signatures of positive selection, and investigate MHC disease associations in different species (44–46). Most researchers are currently using microsatellite repeats and microsatellite SNP combinations as a source of more rapidly diversifying gene markers to provide a better understanding of MHC evolution and function (47).

Compared with other mammalian species, the MHC class II region in horse shows remarkable high variation of the ELA-DRB, -DQA, -DQB, and -DOB loci (48). Similarly, the number of ELA-DRB genes shows a high variability as in other mammalian species (e.g., two or three DRB genes reported in domestic horses and two in Przewalski horses) (33, 49, 50). There is hardly any sequence instruction found of URRs for class II genes in horses, and almost no reports on the genetic control of ELA-DRB gene expression. On the other hand, 27 haplotypes, defined as “COR” haplotypes, were reported in horses with unknown serotypes (negative for known ELA serotypes) (27).

In general, Arabian horses are one of the oldest recognized horse breeds worldwide (51, 52). Unfortunately, only a few molecular or evolutionary studies of Arabian horse populations have been published (13, 53). In addition, it has been suggested that high levels of genetic diversity (54) or reduced genetic variation (55) depend on the population studied. Furthermore, some studies have disputed the accuracy of the Arabian horse pedigrees (53, 56–58) and even the ancient origin of the breed (59). Prior to 1950, most Arabian horse pedigrees were maintained only by oral tradition, and no verified early written pedigrees have been preserved.

Previous studies of the equine MHC that included Arabian horses used antisera that recognized only a small number of MHC haplotypes (60, 61), molecular hybridization specific for only a few individual MHC genes (38), or the previously reported panel of 12 intra-MHC microsatellites (62, 63). Only 9 of the 50 microsatellite-defined haplotypes were detected in the 32 Arabian horses studied, and the provenance of those horses was not defined (27). In this study, we employed a molecular genetic approach that incorporated the analysis of MHC homozygous Arabian horses using the ELA system. We applied fragment length analysis (FLA) of microsatellite markers distributed within the MHC region to associate specific microsatellite constellations with prevalent MHC haplotypes, thereby improving the resolution of genetic diversity assessment within the equine MHC. Specifically, we examined MHC variation in 57 SE Arabian horses based on a panel of 16 highly polymorphic intra-MHC microsatellite loci.

## 2 Materials and methods

### 2.1 Samples involved in this study

Horse samples for this study were kindly provided by Dr. Hans Nagel (NK Katharinenhof Stud, Lower Saxony, Germany) and Irhama Bin Issa Al Sahlawi (Al Sarat Stud, Doha, Qatar). Samples being provided to the Immunology lab at the University of Veterinary Medicine Vienna represented remnant specimens after routine veterinary health checks and were accompanied by written informed consents of the owners. Thus, specific approval of the Ethics and Animal Welfare Committee to use leftover specimens for research purposes was not required (Good Scientific Practice Guidelines updated 1 July 2019, University of Veterinary Medicine Vienna).

### 2.2 Horse blood samples

The number of SE Arabian horses is 57 horses. They arrived in our lab in two groups. The first group, 18 horses, came from the Katharinenhof farm by Dr. Hans Nagel in Germany. The second group, 39 horses, arrived from the State of Qatar. This group represents most of the genetic population of Egyptian Arabian horses. Blood samples were collected using PAXgene Blood DNA Tubes (PreAnalytiX via Qiagen GmbH, Hilden, Germany). Samples were stored at 4 °C for DNA extraction within the first week of collection, or at −20 °C if DNA extraction was to be performed more than 2 weeks after sample collection.

### 2.3 Isolation of genomic DNA from whole blood

Genomic DNA (gDNA) was isolated from the samples of blood using the PAXgene Blood DNA kit (PreAnalytiX via Qiagen) following the manufacturer’s protocol.

gDNA concentration and quality (260/280 and 260/230 ratios) were determined using the NanoDrop 2000c™ spectrophotometer (Thermo Fisher Scientific, Waltham, MA, United States) in pedestal mode (**Supplementary Table 1** in Online Resource 1).

### 2.4 Equine MHC microsatellite amplification

Fluorescently labeled PCR primers for 16 equine intra-MHC polymorphic microsatellites (**Table 1**) were used to amplify fragments from each horse. All 16 markers had been previously identified (11, 63). The 16 markers are located throughout the equine MHC, with each class of MHC I, MHC II, and MHC III.

**Table 1:** Details of microsatellites used for the evaluation of MHC diversity in Straight Egyptian Arabian horses.

Microsatellite locus	MHC region	TB-T2T location (bp) *
UMNJH38	I	31963062–31963215
COR110	I	32020027–32020154
TAMU305-93	I	32020027–32020362
CZM001	I	33130300–33130476
CZM002	I	33163382–33163630
TKY2933	I	33808252–33808467
ABGe9019	III	34705995–34706286
UMNe65	III	34795735–34795982
ABGe9030	II	35970339–35970549
EQMHC1	II	36115633–36115816
CZM004	II	36115641–36115760
TKY3324	II	36569077–36569320
COR112	II	36701425–36701677
COR113	II	36900070–36900333
UM011	II	36929381–36929544
COR114	II	36935556–36935802

*Position of the first nucleotide of each microsatellite repeat motif on TB-T2T GenBank assembly (No. GCA_041296265.1).

Six markers were assigned to the MHC class I gene cluster (UMNJH38, COR110, TAMU305-93, CZM001, CZM002, and TKY2933), two markers were assigned to the MHC class III region (ABGe9019 and UMNe65), and eight markers were assigned to the MHC class II region (ABGe9030, EQMHC1, CZM004, TKY3324, COR112, COR113, UM011, and COR114). Marker localization and genomic context were evaluated using the current equine reference genome of the TB-T2T assembly coordinates (Accession number JBFNWK000000000.1) (**Figure 1**; **Table 1**).

The fluorescently labeled polymerase chain reaction (PCR) primers used in this study are listed in **Supplementary Table 2** in Online Resource 2. A total amount of 50 to 70 ng of gDNA was added to a 12.5-μL reaction mix, containing 1× Type-it^®^ Multiplex PCR Master Mix (Qiagen GmbH) and 0.5–0.6 μM fluorescent-labeled forward primers (Eurofins Genomics GmbH, Ebersberg, Germany). Amplification was carried out under the following cycling conditions: 1 cycle of 95 °C for 5 min, followed by 35 cycles of 95 °C for 30 s, 60 °C for 30 s, and 72 °C for 30 s, and 1 cycle of 72 °C for 10 min for the final extension.

For quality control, PCR reactions were size separated using the QIAxcel Connect System capillary electrophoresis analyzer with the QIAxcel DNA High Resolution Kit and the QX Alignment Marker 15 bp/1,000 bp (Qiagen GmbH). The presence and size of obtained PCR products were accurately determined using QIAxcel ScreenGel Software (Qiagen GmbH).

### 2.5 Fragment length analysis

PCR fragments were size separated on an ABI sequencing device (Eurofins Genomics GmbH). DNA sizing and quality allele calling for the analyzed microsatellite loci were carried out using GeneMapper™ Software (Thermo Fisher Scientific Inc.).

### 2.6 Statistical analysis

For statistical analysis, we have used the software GenAlEx (version number 6.5) being implemented in Microsoft Excel to calculate the following parameters:

Observed heterozygosity (Ho): It shows the individual ratio within the population that are heterozygous at a specific locus, indicating the existing genetic variation in the studied group. In addition, it supplies the actual genetic diversity directly and compares with the expected heterozygosity to infer inbreeding, population structure, or selection.

Expected heterozygosity (He): It measures the possible differences of two random selected allele of a population, suggesting Hardy–Weinberg equilibrium (HWE), which reflects the potential genetic diversity. Moreover, it is considered as a guideline to compare with observed heterozygosity.

Unbiased expected heterozygosity (uHe) is a sample size corrected estimate of expected heterozygosity that reduces bias in small or uneven populations. It is statistically important because it reflects a more accurate measure of genetic diversity, especially when comparing populations with different sample sizes.

Fixation index (*F*): It measures in a population the degree of deviation between expected heterozygosity and observed heterozygosity. It indicates the degree of inbreeding among genetically heterogeneous individuals. Its statistical significance lies in providing a direct explanation of mating patterns, population structure, and departures from the HWE.

The PIC indicates the informative value of a genetic marker. This is measured by examining the number of alleles and their frequency distribution within a population. Its statistical significance lies in the fact that a high value indicates a greater discriminatory ability of the marker, which is used in analyzing diversity and inferring population genetic characteristics.

HWE: This criterion describes the estimated allocation of genotype distribution, assuming random mating and the absence of selection, migration, or genetic drift. It is statistically important because it reveals underlying biological processes such as population substructure, inbreeding, and selection acting on the locus.

Analysis of molecular variance (AMOVA) is used to break down total genetic variance into components attributable to differences between and within populations. Among populations, variance component was calculated, and among individuals within populations and within individuals we used $\sigma_{AP}^{2}$.

The parameter fixation index (*F*_st_) is a measure of genetic differentiation between populations. It represents the proportion of total genetic variation resulting from differences in allele frequencies between populations.

For a one-level AMOVA: $F_{\text{st}}=\frac{\sigma_{AP}^{2}}{\sigma_{Total}^{2}}$ Finally, the principal coordinates analysis (PCoA) is used to visualize genetic relationships between individuals and populations based on genetic distance matrices.

## 3 Results

### 3.1 Genetic diversity of MHC-linked microsatellite markers in the SE Arabian horse in both studied populations

Sixteen microsatellite loci located on MHC I, II, and III regions were tested. The data showed the number of detected alleles for each locus ranging from 4 (COR113) to 12 (CZM002 and ABGe9030), with an average allelic richness of 7.88 alleles. However, the size of allele varied considerably between loci, showing the locus-specific variability of the analyzed population (**Table 2**).

**Table 2:** Heterozygosity analysis of MHC microsatellite markers in all studied Straight Egyptian Arabian horses.

Microsatellite locus	MHC region	TB-T2T location (bp)	Number of detected alleles	Allele size range	Hobs	Hexp	uHe	*F*	PIC
UMNJH38	I	31963062	8	130–155	0.561	0.598	0.603	0.061	0.463
COR110	I	32020027	9	196–211	0.737	0.831	0.839	0.114	0.810
TAMU305-93	I	32020027	10	331–345	0.596	0.850	0.857	0.298	0.856
CZM001	I	33130300	7	179–185	0.561	0.793	0.800	0.292	0.843
CZM002	I	33163382	12	202–255	0.860	0.877	0.885	0.020	0.881
TKY2933	I	33808252	8	199–219	0.404	0.542	0.547	0.256	0.378
ABGe9019	III	34705995	10	288–309	0.554	0.789	0.796	0.298	0.746
UMNe65	III	34795735	6	247–252	0.608	0.766	0.774	0.207	0.721
ABGe9030	II	35970339	12	196–224	0.772	0.849	0.857	0.091	0.827
EQMHC1	II	36115633	7	180–185	0.684	0.685	0.692	0.002	0.597
CZM004	II	36115641	6	114–126	0.298	0.570	0.575	0.476	0.419
TKY3324	II	36569077	5	245–254	0.070	0.614	0.620	0.886	0.488
COR112	II	36701425	8	241–256	0.737	0.785	0.792	0.062	0.817
COR113	II	36900070	4	260–264	0.439	0.562	0.567	0.220	0.415
UM011	II	36929381	7	159–176	0.649	0.797	0.804	0.185	0.787
COR114	II	36935556	7	223–238	0.632	0.761	0.768	0.170	0.707
Mean	–	–	7.875	–	0.573	0.729	0.736	0.227	0.672

Hobs, observed heterozygosity; Hexp, expected heterozygosity; uHe, unbiased expected heterozygosity; *F*, fixation index and values of *F*: ~0; random mating greater than 0, heterozygote deficiency, PIC, polymorphism information content <0.25, low informativeness; 0.25–0.50, moderate; >0.5, highly informative (mark every locus higher than 0.5, and focus on the importance of it).

Ho values showed apparent variation in loci, ranging from 0.070 at TKY3324 to 0.860 at CZM002. He values were persistently higher than Ho at most loci, ranging from 0.542 (TKY2933) to 0.877 (CZM002). The mean Ho and He values of all loci were 0.573 and 0.729, respectively. Accordingly, the mean uHe was detected at 0.736, reflecting the He values and considering the sample size effects (**Table 2**).

*F* values of all loci except for ABGe9030 and EQMHC1 showed heterozygote deficiency (*F* > 0) with a wide variation among loci, ranging from 0.002 (EQMHC1) to 0.886 (TKY3324). The mean *F* value of loci was 0.227. PIC values of most loci are highly informative (PIC > 0.5), whereas UMNJH38, TKY2933, CZM004, TKY3324, and COR113 are moderately informative (PIC ranging from 0.25 to 0.5). PIC values ranged from 0.378 to 0.881, with the highest values observed at highly polymorphic loci such as CZM002 and TAMU305-93. The mean PIC of all loci was 0.672, suggesting moderate to high novelty of the analyzed MHC microsatellite markers of the studied population (**Table 2**).

### 3.2 Genetic diversity parameters following subdivision of the SE Arabian horse population into two groups

The data showed that the detected number of alleles per locus in the AR-DE group from Germany ranged from 2 to 9. However, Ho values of loci varied between 0.111 (TKY2933) and 1.000 (CZM002), with a mean of Ho (0.604). He values ranged from 0.105 to 0.801, giving a mean He value of 0.651. F values of UMNJH38, COR110, CZM001, ABGe9019, UMNe65, TKY3324, and COR112 showed heterozygote deficiency (*F* > 0.0), whereas loci CZM001, TKY2933, ABGe9030, EQMHC1, CZM004, COR113, and UM011 showed heterozygote excess (*F* < 0.0). Only COR114 exhibited random mating (*F* = 0.0). The *F* values for this group showed a wide range from −0.371 (EQMHC1) to 0.682 (ABGe9019), with a mean *F* value of 0.061. All loci were highly informative (PIC > 0.5), except EQMHC1 and CZM004, which were moderately informative (PIC 0.25 to 0.5), and TKY2933 and COR112, which were poorly informative (PIC < 0.25), respectively. In summary, the PIC variations of loci suggest differences in allelic distribution and heterozygosity within this subgroup (**Table 3**).

**Table 3:** Heterozygosity analysis of MHC microsatellite markers of the two studied populations of Straight Egyptian Arabian horses.

Microsatellite locus	MHC region	AR-DE						AR-QA					
		NA	Hobs	Hexp	*F*	PIC	HWE	NA	Hobs	Hexp	*F*	PIC	HWE
UMNJH38	I	6	0.444	0.659	0.326	0.561	***	5	0.615	0.559	−0.102	0.402	***
COR110	I	7	0.722	0.772	0.064	0.722	ns	7	0.744	0.801	0.072	0.778	ns
TAMU305-93	I	8	0.722	0.789	0.084	0.772	***	8	0.538	0.823	0.346	0.823	***
CZM001	I	5	0.722	0.758	0.047	0.876	***	7	0.487	0.768	0.366	0.783	***
CZM002	I	9	1.000	0.801	−0.249	0.764	ns	10	0.795	0.853	0.068	0.866	ns
TKY2933	I	2	0.111	0.105	−0.059	−0.054	ns	8	0.538	0.672	0.199	0.579	ns
ABGe9019	III	5	0.222	0.699	0.682	0.617	***	9	0.711	0.770	0.077	0.720	*
UMNe65	III	5	0.722	0.742	0.027	0.680	ns	4	0.545	0.696	0.216	0.629	ns
ABGe9030	II	7	0.833	0.789	−0.057	0.747	***	10	0.744	0.833	0.107	0.805	*
EQMHC1	II	5	0.833	0.608	−0.371	0.479	ns	6	0.615	0.702	0.123	0.621	***
CZM004	II	3	0.500	0.495	−0.009	0.306	***	5	0.205	0.596	0.656	0.460	***
TKY3324	II	4	0.222	0.647	0.656	0.542	***	3	0.000	0.440	1.000	0.225	***
COR112	II	6	0.778	0.787	0.012	0.846	ns	6	0.718	0.720	0.002	0.716	ns
COR113	II	2	0.222	0.198	−0.125	−0.032	ns	4	0.538	0.618	0.129	0.510	ns
UM011	II	6	0.833	0.792	−0.053	0.754	ns	5	0.564	0.697	0.191	0.669	*
COR114	II	5	0.778	0.778	0.000	0.731	**	5	0.564	0.649	0.131	0.541	ns
Mean	–		0.604	0.651	0.061	0.582			0.581	0.675	0.142	0.633	

AR-DE, Straight Egyptian Arabian horse from Germany; AR-QA, Straight Egyptian Arabian horse from Qatar; NA, number of detected alleles; Hobs, observed heterozygosity; Hexp, expected heterozygosity; uHe, unbiased expected heterozygosity; *F*, fixation index and values of *F*: ~0; random mating greater than 0, heterozygote deficiency less than 0, heterozygote excess, PIC: polymorphism information content <0.25, low informativeness; 0.25–0.50, moderate; >0.5, highly informative (mark every locus higher than 0.5, and focus on the importance of it) and HWE, a departure from the Hardy–Weinberg equilibrium (*p < 0.05, **p < 0.01, ***p < 0.001).

The data of the AR-QA group from Qatar showed that the allelic diversity per locus ranged from 3 to 10 alleles. Ho values ranged from 0.000 (TKY3324) to 0.795 (CZM002), whereas He values ranged from 0.440 to 0.853. The mean Ho and He values were 0.581 and 0.675, respectively. *F* values of most loci showed heterozygote deficiency (*F* > 0.0) except for UMNJH38, which exhibited heterozygote excess (*F* < 0.0). COR112 showed random mating (*F* = 0.0). *F* values ranged from −0.102 to 1.000, and the mean *F* value was 0.142. The mean *F* value in this group was 0.142, ranging from −0.102 to 1.000. All loci were highly informative (PIC > 0.5) except for UMNJH38 and CZM004, which were moderately informative (PIC 0.25 to 0.5). TKY3324 showed low informativeness (PIC < 0.25). Similar to the AR-DE group, PIC variations of loci exhibited differences in allelic distribution and heterozygosity within this subgroup (**Table 3**). In both subpopulations, PIC values varied by locus and population, suggesting locus-specific patterns of genetic variability within each group (**Table 3**).

Several MHC microsatellite loci showed significant deviations from the HWE in both studied populations (AR-DE and AR-QA). On the one hand, in the AR-DE group, 8 out of 15 loci deviated significantly from the HWE (*p* < 0.05), including UMNJH38, TAMU305-93, CZM001, ABGe9019, ABGe9030, CZM004, TKY3324, and COR114. Most of these loci showed either heterozygote deficiency (positive *F* values, e.g., ABGe9019, *F* = 0.682; TKY3324, *F* = 0.656) or excess (negative *F* values, e.g., ABGe9030, *F* = −0.057; UMNJH38, *F* = 0.326). The mean fixation index across loci *F* was 0.061, indicating a slight overall heterozygote deficit (**Table 3**).

On the other hand, in the AR-QA population, 9 of 15 loci showed a significant deviation from the HWE: UMNJH38, TAMU305-93, CZM001, ABGe9019, ABGe9030, EQMHC1, CZM004, TKY3324, and UM011. A marked heterozygote deficiency was observed at TKY3324 (*F* = 1.000) and CZM004 (*F* = 0.656). The mean *F* was higher in AR-QA (*F* = 0.142), implying a more evident deviation from HWE compared to AR-DE. In general, both populations showed multiple departures from HWE across MHC loci, with AR-QA showing slightly more affected loci and a higher average *F* (**Table 3**).

### 3.3 AMOVA analysis of distribution of molecular variance

Most of the molecular variation was distributed within individuals (57%), followed by variation among individuals (42%), whereas variation among populations accounted for 0% of the total variance (**Table 4**). Almost all genetic variations were found within individuals and among individuals within populations, while none is attributable to differences between the two populations (AR-DE vs. AR-QA) as shown below.

**Table 4:** Analysis of molecular variance (AMOVA) between AR-DE and AR-QA Arabian horse populations.

Source	df	SS	MS	Est. Var.	%
Among Pops	1	12.769	12.769	0.030	0%
Among Indiv	55	621.064	11.292	3.374	42%
Within Indiv	57	259.000	4.544	4.544	57%
Total	113	892.833		7.948	100%

df, degrees of freedom; SS, sum of squares; MS, mean squares; Est. Var., estimated variance.

#### 3.3.1 Among populations

Variance component $\sigma_{AP}^{2}=0.030$, percentage $\frac{0.030}{7.948}\times 100=0.38\%$, rounded by GenAlEx to  0%. Only approximately 0.4% of total MHC microsatellite variation was explained by differences between the German and Qatari breeding populations (extremely low), suggesting that AR-DE and AR-QA are genetically very similar at these MHC loci, providing little evidence of population subdivision, and implying that both breeding programs likely originate from a common genetic pool and have maintained similar MHC diversity. Genetic differentiation among populations was given by the fixation index (*F*_st_ = 0.004) (**Table 5**).

**Table 5:** Fixation indices and significance values based on one-level AMOVA.

*F*-statistics	Value	*P*(rand ≥ data)
*F*_st_	0.004	0.009
*F*_is_	0.426	0.001
*F*_it_	0.428	0.001

*F*_st_, genetic differentiation among populations; *F*_is_, inbreeding coefficient within populations; *F*_it_, overall inbreeding coefficient relative to the total population. Significance was assessed using 999 permutations. *F*_st_ *<*0.01, negligible differentiation; 0.01–0.05, low; 0.05–0.15, moderate; >0.15, strong.

#### 3.3.2 Among individuals within populations

Variance component $\sigma_{AP}^{2}=3.374$, percentage $\frac{3.374}{7.948}\times 100=42\%$. As expected in microsatellite datasets, 42% of variation occurs because individuals within the same breeding population differ from one another, indicating that most diversity is maintained among horses rather than between breeding programs. Individual horses possess distinct MHC haplotypes/genotypes.

#### 3.3.3 Within individuals

Variance component $\sigma_{AP}^{2}=4.544$, percentage $\frac{4.544}{7.948}\times 100=57\%.$This is the largest component for codominant simple sequence repeats (SSRs) in diploids, and this component reflects heterozygosity. High within-individual variation generally indicates (i) a substantial heterozygosity, and (ii) multiple alleles maintained in the population. For MHC-linked loci, this can be consistent with the maintenance of immunogenetic diversity.

### 3.4 Genetic relationships between individuals and populations

**Figure 2:**
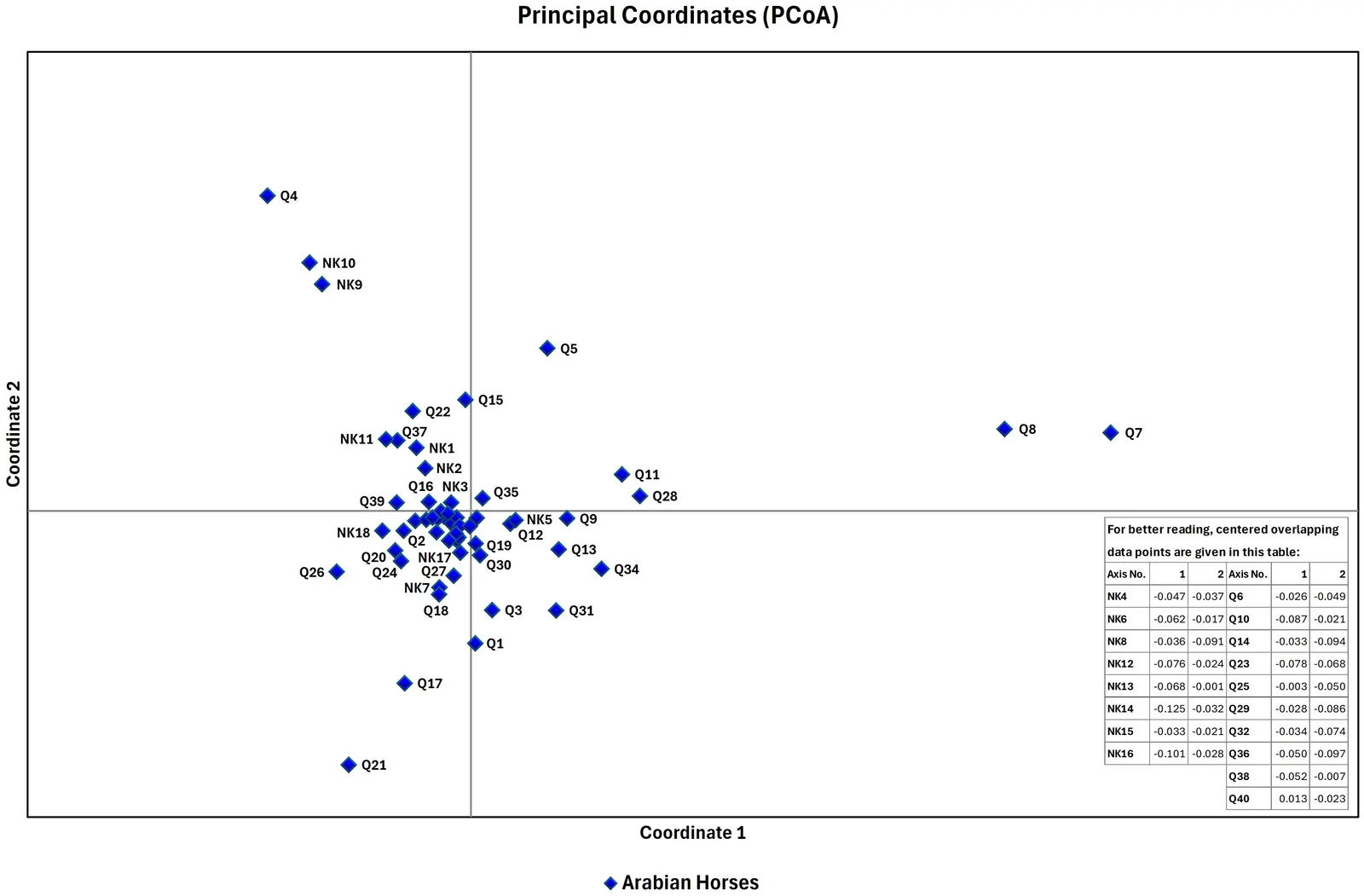
Principal coordinates analysis (PCoA) showing genetic relationships among Straight Egyptian Arabian horses based on MHC-linked microsatellite markers. It demonstrates that most individuals clustered closely together, and there is no clear separation between the two populations. Only Q7, Q8, Q21, Q4, NK9, and NK10 showed more distant from the main cluster, indicating genetic variation rather than strong differentiation.

The results of the PCoA showed a strong overlap between many individuals except for those which can be of interest and to be analyzed further (Q4, NK10, NK9, Q5, Q8, and Q7). Additionally, it is notable that majority of AR-DE individuals overlap with AR-QA, suggesting that there is no obvious influence of different breeding program strategies or negligible differentiation between them (**Table 6**; **Figure 2**).

**Table 6:** Principal coordinate analysis (PCoA).

Percentage of variation explained by the first 3 axes			
Axis	1	2	3
%	2.97	2.92	2.75
Cum %	2.97	5.89	8.65

## 4 Discussion

Although the SE Arabian horses represent less than 2% of all registered Arabians (64), the genetic diversity collected in the present study for MHC-linked microsatellite markers is largely comparable to those published in other studies on Arabian and other horse populations (62, 63). However, our data show a higher number of some detected alleles than a previous study of Persian Arabian horses (UMNJH38, 8 and 4, respectively). Our data also show a similar pattern to previous studies of Persian Arabian horses and Icelandic horses regarding the high levels of observed and expected heterozygosity in most MHC-linked loci (62). Previous reports on Persian Arabian horses and Icelandic horses claimed a correspondence between Ho and He values for several examined markers, pointing to a high probability of heterozygous individuals of the MHC region. However, they reported a clearer discrepancy between Ho and He at several loci, suggesting positive *F* values. Conversely, in this study, the SE Arabian horses analyzed show moderate to high Ho and He values overall; however, a more obvious discrepancy between observed and expected heterozygosity has been detected at several loci, resulting in positive fixation index values. Our results showed a less homogeneous distribution of heterozygosity in MHC-linked loci compared to published studies, which consistently claimed high diversity levels.

Regarding the PIC and the number of detected alleles, our data show that the range has been reported in previous studies using expanded microsatellite panels to analyze MHC diversity in multiple horse breeds. They showed that MHC class I, II, and III regions widely exhibited moderate to high PIC values. Although considerable variation exists in loci depending on allele frequency distribution in populations, our sample of the SE Arabian horse population retains noticeable MHC-associated genetic variation, as has been confirmed by moderate to high PIC values and allelic diversity at most loci, although with greater locus-to-locus variability than that reported in some Arabian and isolated horse populations. This comparison highlights that SE Arabian horses exhibit reasonable levels of genetic diversity associated with immunity. These levels are very close to those of other local horse breeds, with a more varied pattern of microsatellite marker diversity. The comparatively high average He and PIC values of loci indicate the suitability of these markers for evaluating genetic diversity and population structure.

He exceeded Ho at most loci, indicating positive *F* at the population level. This pattern shows a deviation from HWE in multiple loci. This suggests a non-random mating or the influence of inbreeding strategies. The broad range of *F* values emphasizes locus-specific differences in allelic allocation. Because of the evolutionary mechanisms underlying MHC markers, this feature is commonly observed in them.

The results of the analysis of the two groups showed similar levels of genetic diversity. Although there were differences in some parameters, such as heterozygosity and *F* value, between the two groups, both groups have fair levels of heterozygosity and allelic diversity. Higher *F* values in AR-QA were demonstrated at certain loci, reflecting a greater lack of heterozygosity at those loci. This shows differences in breeding history, founder influences, or breeding management practices. In contrast, the lower mean *F* value in AR-DE compared to AR-QA indicates a closer match between the Ho and He results.

Polymorphism is high at many loci in both populations. This highlights that genetic variation in the MHC region remains conserved in different breeding contexts. The maintenance of highly characterizing loci in both groups indicates the strength of the microsatellite markers used to examine genetic variation in functionally important immune-related regions. However, loci with lower heterozygosity and PIC values in one or both groups may lead to skewed allele frequencies and reduced allelic diversity, emphasizing the heterogeneous nature of MHC-linked variation.

It seems that evolutionary and population-level leads to deviation from equilibrium at several MHC loci in both AR-DE and AR-QA genomic regions. This might be explained by the fact that the markers are in the MHC region, which is functionally linked to immune response, separated from HWE, which may suggest that the small increase in the mean *F* in AR-QA compared to AR-DE implies a greater lack of heterozygosity. This pattern could reflect a breeding system based on inbreeding, a small effective population size, or the intensive use of closely related breeding strains. However, AR-DE showed fewer extreme *F* values and less overall deviation, indicating a relatively greater genetic balance at these loci.

Specific genetic loci, such as TKY3324 and CZM004, showed a marked decrease in the number of homozygous individuals in both groups, or it may be the Wahlund effect, which is the apparent decrease in Ho when genetically distinct subgroups are analyzed as a single group. However, CZM002 and COR112 loci remain in equilibrium in both groups, indicating a balanced distribution of alleles and possibly neutral behavior for these markers.

Distinguishing between MHC class I and class II variation is of biological importance. MHC class I variation may affect the presentation of intracellular pathogen-derived peptides to CD8^+^ cytotoxic T cells, which are essential for antiviral responses. MHC class II variation may affect antigen presentation to CD4^+^ helper T cells. Because the current panel of markers covers both classes I and II, the observed variation provides an indirect overview of immunological variation. Heterozygosity in MHC-associated markers is important. Individuals with high heterozygosity carry a wider range of MHC phenotypes, potentially increasing the range of peptides that can be presented to T cells. Conversely, a significant lack of heterozygosity leads to a decrease in the number of phenotypes. These phenotypes are particularly important for selectively managed, small-population horse breeds, where breeding decisions influence the maintenance of immunologically related genetic variation.

The high allelic diversity, high He, and high PIC values indicate that the population retains multiple haplotype backgrounds. This haplotype diversity may be beneficial because the MHC I and MHC II genes encode molecules responsible for antigen presentation. Therefore, the broad genetic background associated with MHC may influence the recognition of pathogenic peptides more broadly. However, this relationship remains indirect and requires sequencing-based MHC typing and functional immunoassays for confirmation.

In conclusion, the recorded patterns of genetic diversity within and between the studied groups are compatible with the complex structure and evolutionary importance of the ELA region. These observations provide baseline data on MHC-associated genetic diversity in SE Arabian horses. It might be considered a foundation for future studies examining population structure, breeding strategies, and the maintenance of immunogenetic variation in the Arabian horse. The HWE results indicate biologically relevant differences in genetic structure between the two SE Arabian horse groups.

## Data Availability

The raw data supporting the conclusions of this article will be made available by the authors, without undue reservation.
