# Correction: First-time description of MHC diversity in Straight Egyptian Arabian horses using immune-linked microsatellite markers

**DOI:** 10.3389/fimmu.2026.1980225

**Published:** 2026-09-25

**Authors:** Abdullah Saleh A. Alkhamees, Jessika-M. V. Cavalleri, Sabine E. Hammer

**Affiliations:** 1 Immunology Unit, Centre of Pathobiology, Department of Biological Sciences and Pathobiology, University of Veterinary Medicine Vienna, Vienna, Austria; 2 Equine Internal Medicine, Clinical Centre for Equine Health and Research, Clinical Department of Small Animals and Horses, University of Veterinary Medicine Vienna, Vienna, Austria

**Keywords:** ELA, equine leukocyte antigen, major histocompatibility complex, MHC diversity, microsatellite analysis

The open access **Funding** information “Open access funding provided by University of Veterinary Medicine Vienna” was erroneously omitted from the funding statement.

**Funding** statement for original article:

“The author(s) declared that financial support was received for this work and/or its publication. The present study was funded by King Faisal University through the Saudi Arabian Cultural Mission in Austria. The sponsor was not involved in the study design, collection, analysis, interpretation of data, the writing of this article, or the decision to submit it for publication. Open access funding provided by University of Veterinary Medicine Vienna.

The original version of this article has been updated.

