# Supplementary material for: First-time description of MHC diversity in Straight Egyptian Arabian horses using immune-linked microsatellite markers
Source: Front Immunol. 2026 Aug 17;17:1859977. doi: 10.3389/fimmu.2026.1859977 (PMC13527931; doi:10.3389/fimmu.2026.1859977)
Supplement: Supplementary file 1 [file DataSheet1.pdf]

Supplementary Material

First-time description of MHC diversity in Straight Egyptian Arabian Horses using immune-linked microsatellite markers

Abdullah Saleh A. Alkhamees[1], Jessika-M. Cavalleri[2], Sabine E. Hammer[1*]

[1]Immunology Unit, Centre of Pathobiology, Department of Biological Sciences and Pathobiology, University of Veterinary Medicine Vienna, Vienna, Austria

[2]Equine Internal Medicine, Clinical Centre for Equine Health and Research, Clinical Department of Small Animals and Horses, University of Veterinary Medicine Vienna, Vienna, Austria

*** Correspondence:**
Corresponding Author
sabine.hammer@vetmeduni.ac.at

Keywords: major histocompatibility complex, equine leukocyte antigen, ELA, microsatellite analysis, MHC diversity

1 Supplementary Tables

Supplementary Table 1. Genomic DNA concentrations.

Sample ID	Nucleic Acid	Unit	A260 (Abs)	A280 (Abs)	260/280	260/230
Q1	118.2	ng/µl	2.363	1.221	1.94	2.28
Q2	126.1	ng/µl	2.522	1.290	1.95	2.10
Q3	129.6	ng/µl	2.592	1.338	1.94	2.19
Q4	134.6	ng/µl	2.692	1.407	1.91	2.14
Q5	102.7	ng/µl	2.054	1.056	1.94	2.11
Q5	104.7	ng/µl	2.094	1.075	1.95	2.14
Q6	120.4	ng/µl	2.407	1.245	1.93	2.19
Q7	91.8	ng/µl	1.836	0.934	1.97	1.58
Q8	122.2	ng/µl	2.444	1.271	1.92	2.27
Q9	117.6	ng/µl	2.352	1.210	1.94	2.02
Q10	113.3	ng/µl	2.266	1.165	1.94	2.20
Q11	107.6	ng/µl	2.151	1.108	1.94	2.21

Q12	124.0	ng/µl	2.480	1.286	1.93	2.16
Q13	111.3	ng/µl	2.227	1.151	1.93	2.17
Q14	113.2	ng/µl	2.264	1.154	1.96	2.17
Q15	101.0	ng/µl	2.020	1.033	1.96	2.25
Q16	105.8	ng/µl	2.117	1.102	1.92	2.20
Q17	87.3	ng/µl	1.745	0.894	1.95	2.22
Q18	113.4	ng/µl	2.269	1.175	1.93	2.24
Q19	103.1	ng/µl	2.063	1.065	1.94	2.24
Q20	123.9	ng/µl	2.478	1.266	1.96	2.19
Q21	106.6	ng/µl	2.133	1.101	1.94	2.25
Q22	101.3	ng/µl	2.026	1.045	1.94	2.27
Q23	96.1	ng/µl	1.923	0.975	1.97	2.23
Q24	103.6	ng/µl	2.072	1.052	1.97	2.12
Q25	97.2	ng/µl	1.944	0.993	1.96	2.24
Q26	90.9	ng/µl	1.818	0.920	1.98	2.14
Q27	96.5	ng/µl	1.930	0.978	1.97	2.17
Q28	97.4	ng/µl	1.948	0.999	1.95	2.18
Q29	92.4	ng/µl	1.848	0.947	1.95	2.24
Q30	110.6	ng/µl	2.212	1.128	1.96	2.24
Q31	116.2	ng/µl	2.324	1.202	1.93	2.22
Q32	107.6	ng/µl	2.151	1.099	1.96	2.17
Q34	101.5	ng/µl	2.030	1.039	1.95	2.28
Q35	108.5	ng/µl	2.170	1.116	1.95	2.19
Q36	100.2	ng/µl	2.004	1.037	1.93	2.28
Q37	100.7	ng/µl	2.014	1.028	1.96	2.22
Q38	92.3	ng/µl	1.845	0.945	1.95	2.16
Q39	111.7	ng/µl	2.235	1.142	1.96	2.28
Q40	116.2	ng/µl	2.323	1.201	1.93	2.28
NK 1	90.0	ng/µl	1.799	0.943	1.91	2.07
NK 2	117.7	ng/µl	2.354	1.231	1.91	2.30
NK 3	98.9	ng/µl	1.978	1.043	1.90	2.28
NK 4	96.5	ng/µl	1.930	1.002	1.93	2.14
NK 5	96.3	ng/µl	1.927	1.011	1.91	2.12
NK 6	87.0	ng/µl	1.740	0.905	1.92	2.23
NK 7	106.9	ng/µl	2.138	1.121	1.91	2.12
NK 8	116.4	ng/µl	2.328	1.237	1.88	1.98
NK 9	101.3	ng/µl	2.027	1.067	1.90	2.07

NK 10	129.5	ng/µl	2.589	1.354	1.91	2.30
NK 11	89.4	ng/µl	1.788	0.934	1.91	2.28
NK 12	97.3	ng/µl	1.946	1.018	1.91	2.29
NK 13	121.4	ng/µl	2.428	1.278	1.90	2.22
NK 14	104.3	ng/µl	2.085	1.090	1.91	2.24
NK 15	132.7	ng/µl	2.653	1.400	1.89	2.28
NK 16	117.7	ng/µl	2.354	1.236	1.90	2.27
NK 17	100.6	ng/µl	2.011	1.059	1.90	2.19
NK 18	71.1	ng/µl	1.421	0.741	1.92	2.30

Q: Straight Egyptian Arabian Horse from Qatar (AR-QA).
NK: Straight Egyptian Arabian horse from Germany (AR-DE).

Supplementary Table 2. Primers Sequences used for PCR amplification of the target loci.

MHC class	Marker ID	5' to 3'	5' dye label
I	UMNJH-38	TGTGTGTGCACCTGTCCTTT	FAM
I	UMNJH-38	GATGGGAGGGAATGAGGAAT	
I	COR110	TTTGGTCTTTGCAGGTATGG	Yakima Yellow
I	COR110	TCTCCCTTCCTCTTTGTTCC	
I	TAMU305-93	GAAGCCCAGTCTGAGTGAAGAT	ATTO 550
I	TAMU305-93	ATAAGACTGTAGCTACCTGTCCTCA	
I	CZM002	GCACTGAGCAAGTTAAGGAGAC	FAM
I	CZM002	GACTGCATCAGGTCAGGTCA	
I	CZM001	AGGCTGCACACTTTCAGTTATAA	ATTO 550
I	CZM001	ACTATTCCCTCTGGCTCAGTTAC	
I	TKY2933	GATCTTCAATCTCCACACAAGC	ATTO 565
I	TKY2933	CTTAGTGGCCACTCAGTGATTT	
III	ABGe9019	CTGAGAGAGACAGCATTTGTGG	FAM
III	ABGe9019	GAAAGGTGTCTCCATTGTTGCT	
III	UMNe65	TCGCAAAACCCACAGACTAC	Yakima Yellow
III	UMNe65	TTCTCCTTTCCTTCCACTCC	
II	ABGe9030	CCAGCAGACCTGCAAGAGTA	FAM
II	ABGe9030	AGCATGAGAGCCATGAAGGT	
II	EQMHC1	ATGCATACCGGGAAAGACAG	Yakima Yellow
II	EQMHC1	AGAGACTTCAGTCTCTGTGGTG	

II	COR112	TTACCTGGTTATTGGTTATTTGG	ATTO 550
II	COR112	TCACCCACTAAATCTCAAATCC	
II	COR113	TGTTTAGAACTCGCCAGGAG	ATTO 565
II	COR113	TCATCAGTTCCTTGCCTAGC	
II	UM011	TGAAAGTAGAAAGGGATGTGG	FAM
II	UM011	TCTCAGAGCAGAAGTCCCTG	
II	COR114	TCAAAATCCACACTCCCTTC	Yakima Yellow
II	COR114	TCCATAAAGAGTGGGACACTG	
II	CZM004	CGGGAAAGACAGAGGCTATT	ATTO 550
II	CZM004	GTATATTGATAAAGCCAGGGATTT	
II	TKY3324	AGCCGTCCTGTTCCAACTAA	ATTO 565
II	TKY3324	TGCCCCTTAAAACTCTGTCTTT	

2 References

Horecky C, Horecka E, Futas J, et al. Microsatellite markers for evaluating the diversity of the natural killer complex and major histocompatibility complex genomic regions in domestic horses. HLA. 2018; 91:271–9.

Holmes CM, Violette N, Miller D, et al. MHC haplotype diversity in Icelandic horses determined by polymorphic microsatellites. Genes Immun. 2019; 20:660–70.
